# Prognostic stratification for *IDH*-wild-type lower-grade astrocytoma by Sanger sequencing and copy-number alteration analysis with MLPA

**DOI:** 10.1038/s41598-021-93937-8

**Published:** 2021-07-13

**Authors:** Yasuhide Makino, Yoshiki Arakawa, Ema Yoshioka, Tomoko Shofuda, Takeshi Kawauchi, Yukinori Terada, Masahiro Tanji, Daisuke Kanematsu, Yohei Mineharu, Susumu Miyamoto, Yonehiro Kanemura

**Affiliations:** 1 Department of Neurosurgery, Kyoto University Graduate School of Medicine, Kyoto, 606-8507 Japan; 2 Department of Biomedical Research and Innovation, Institute for Clinical Research, National Hospital Organization Osaka National Hospital, Osaka, 540-0006 Japan; 3 Department of Neurosurgery, National Hospital Organization Osaka National Hospital, Osaka, Japan

**Keywords:** Cancer genetics, Genetic markers

## Abstract

The characteristics of *IDH*-wild-type lower-grade astrocytoma remain unclear. According to cIMPACT-NOW update 3, *IDH*-wild-type astrocytomas with any of the following factors show poor prognosis: combination of chromosome 7 gain and 10 loss (+ 7/− 10), and/or *EGFR* amplification, and/or *TERT* promoter (*TERT*p) mutation. Multiplex ligation-dependent probe amplification (MLPA) can detect copy number alterations at reasonable cost. The purpose of this study was to identify a precise, cost-effective method for stratifying the prognosis of *IDH*-wild-type astrocytoma. Sanger sequencing, MLPA, and quantitative methylation-specific PCR were performed for 42 *IDH*-wild-type lower-grade astrocytomas surgically treated at Kyoto University Hospital, and overall survival was analysed for 40 patients who underwent first surgery. Of the 42 *IDH*-wild-type astrocytomas, 21 were classified as grade 4 using cIMPACT-NOW update 3 criteria and all had either *TERT*p mutation or *EGFR* amplification. Kaplan–Meier analysis confirmed the prognostic significance of cIMPACT-NOW criteria, and World Health Organization grade was also prognostic. Cox regression hazard model identified independent significant prognostic indicators of *PTEN* loss (risk ratio, 9.75; *p* < 0.001) and *PDGFRA* amplification (risk ratio, 13.9; *p* = 0.002). The classification recommended by cIMPACT-NOW update 3 could be completed using Sanger sequencing and MLPA. Survival analysis revealed *PTEN* and *PDGFRA* were significant prognostic factors for *IDH*-wild-type lower-grade astrocytoma.

## Introduction

Glioma is a common tumour type originating in the human brain^1^. Glioblastoma, grade IV (GBM) is the most aggressive and major subtype of glioma, while diffuse astrocytoma, grade II (DA) and anaplastic astrocytoma, grade III (AA) are lower-grade astrocytomas. All these pathological entities had been classified mainly based on histology in the 2007 World Health Organization (WHO) classification of central nervous system tumours^2^. *IDH* mutation is widely recognised as a good predictor of survival among patients with glioma^3^, and codeletion of chromosome 1p and 19q (1p/19q codeletion) has been associated with oligodendroglioma and suggests longer survival^4^. Based on such findings, the WHO 2016 classification combined genetic profiling with a grading system based on the WHO 2007 classification. Oligodendroglioma was defined as a glioma with both *IDH*-mutation and 1p/19q codeletion, and the K27M mutation in *H3F3A* or less common *HIST1H3B* was included in the criteria for diffuse midline glioma, H3K27M-mutant^5^.

*IDH*-mutant and *IDH*-wild-type astrocytomas are well known to show distinct genetic profiles and prognosis^3,6–8^. However, the disease-defining genetic alterations of *IDH*-wild-type astrocytomas have not been revealed^5,9^, and diagnosis remains dependent on the histological findings. However, some studies have concluded that a substantial subset of *IDH*-wild-type lower-grade astrocytomas show a poor survival course similar to that of *IDH*-wild-type GBM^7,9–14^. Based on these studies, a previous report by the Consortium to Inform Molecular and Practical Approaches to CNS Tumour Taxonomy (cIMPACT-NOW) update 3 proposed that certain *IDH*-wild-type diffuse astrocytomas show a poor clinical course similar to *IDH*-wild-type glioblastomas, and that the characteristics of these tumours were a result of at least one of three genetic profiles: combined whole chromosome 7 gain and whole chromosome 10 loss (+ 7/*− *10); and/or *EGFR* amplification; and/or *TERT* promoter (*TERT*p) mutation^15^. These three profiles have been recommended for inclusion in the next classification, with this specific type of astrocytoma to be diagnosed as “*diffuse astrocytic glioma, IDH-wildtype, with molecular features of glioblastoma, WHO grade IV*”, referred to as “*astrocytoma, grade 4*” in the present study. In this manner, copy-number alterations (CNAs) as well as mutations have been studied to allow clear classification of *IDH*-wild-type astrocytoma corresponding to the clinical prognosis.

In general, the hot spot mutations of *IDH1/2* and *TERT*p can be detected by Sanger sequencing^3,16–18^ and 1p/19q codeletion can be examined by fluorescence in situ hybridisation or multiplex ligation-dependent probe amplification (MLPA)^19,20^. MLPA can also detect copy-number alterations in *EGFR* and *PTEN*^19,20^, which are located on chromosome 7p and 10q, respectively. However, whole-chromosomal alterations in chromosomes 7 and 10 were difficult to detect using only one MLPA kit, and are reportedly better ascertained by single nucleotide polymorphism array^11,14^, DNA methylation array^9,13^, array-based comparative genomic hybridisation^14,21^, or next-generation sequencing^12^. Unfortunately, those methods are difficult to conduct in many local institutes and hospitals, and thus are less than ideal as global standards. MLPA can target multiple different sequences in a single PCR reaction and can be used on fragmented DNA for which only small quantities are available (> 20 ng per reaction)^19,22^. The only appliances needed to perform this method are a thermal cycler and a capillary sequencer. MLPA has the potential to detect subgroups of *IDH*-wild-type astrocytoma showing poor prognosis, such as “astrocytoma, grade 4”, but the feasibility of this method needs to be evaluated.

## Results

Between August 19th, 2010, and December 19th, 2019, a total of 291 samples were resected from 257 patients in Kyoto University Hospital and cryopreserved or extracted into DNA before fixation. All samples received integrated diagnosis based on the WHO 2016 classification, and 42 tumours from 42 patients (31 men, 11 women) were classified as IDH-wild-type astrocytoma. The characteristics of these 42 tumours are described in Table 1. The tumours comprised 18 *IDH*-wild-type DAs and 24 *IDH*-wild-type AAs, and median age at resection was 55.5 years (range 5–85 years). No significant differences in age or sex were noted between tumour subtypes. Forty tumours were removed in first surgeries, comprising 18 DAs and 22 AAs, and no difference in age was evident between subtypes. However, initial treatments for AAs included chemoradiation therapy more frequently, whereas observation was selected more in DAs. The initial postsurgical treatments of these patients were 9 chemoradiation therapies and 1 chemotherapy for DAs, and 19 chemoradiation therapies and 1 chemotherapy for AAs, while observation with regular imaging without treatment was selected for 8 DAs and 2 AAs.

**Table 1:** Characteristics of all patients included in the present study.

	Total	DA, *IDH*-wt	AA, *IDH*-wt	p-value*
**All tumours**				
Number, n	42	18	24	
Sex, men, n	31	13	18	1
Age (year), median (range)	55.5 (5–85)	56.5 (5–85)	55 (8–85)	0.964
cIMPACT-NOW update 3 "grade 4", n	21	9	12	1
*TERT*p mutation, n	18	9	9	0.533
*EGFR* amplification, n	8	1	7	0.109
*EGFR* gain and *PTEN* loss, n	6	3	3	1
*PTEN* loss, n	11	3	8	0.299
*PDGFR* amplification, n	4	0	4	0.122
*CDKN2A* homozygous loss, n	8	1	7	0.109
*MGMT*p hypermethylation, n	13	6	7	1
**Patients for survival analysis**				
Number, n	40	18	22	
Age (years), median (range)	57 (5–85)	56.5 (5–85)	59 (8–85)	0.725
**Initial treatment**				
Chemoradiotherapy, n	28	9	19	0.0246
Chemotherapy, n	2	1	1	
Observation, n	10	8	2	

*AA* anaplastic astrocytoma; *DA* diffuse astrocytoma; *IDH*-wt, *IDH* wild type.

*The p-values for comparisons between DA and AA groups are calculated with Student's t-test for age and with Fisher's exact test for the others.

**Figure 1:**
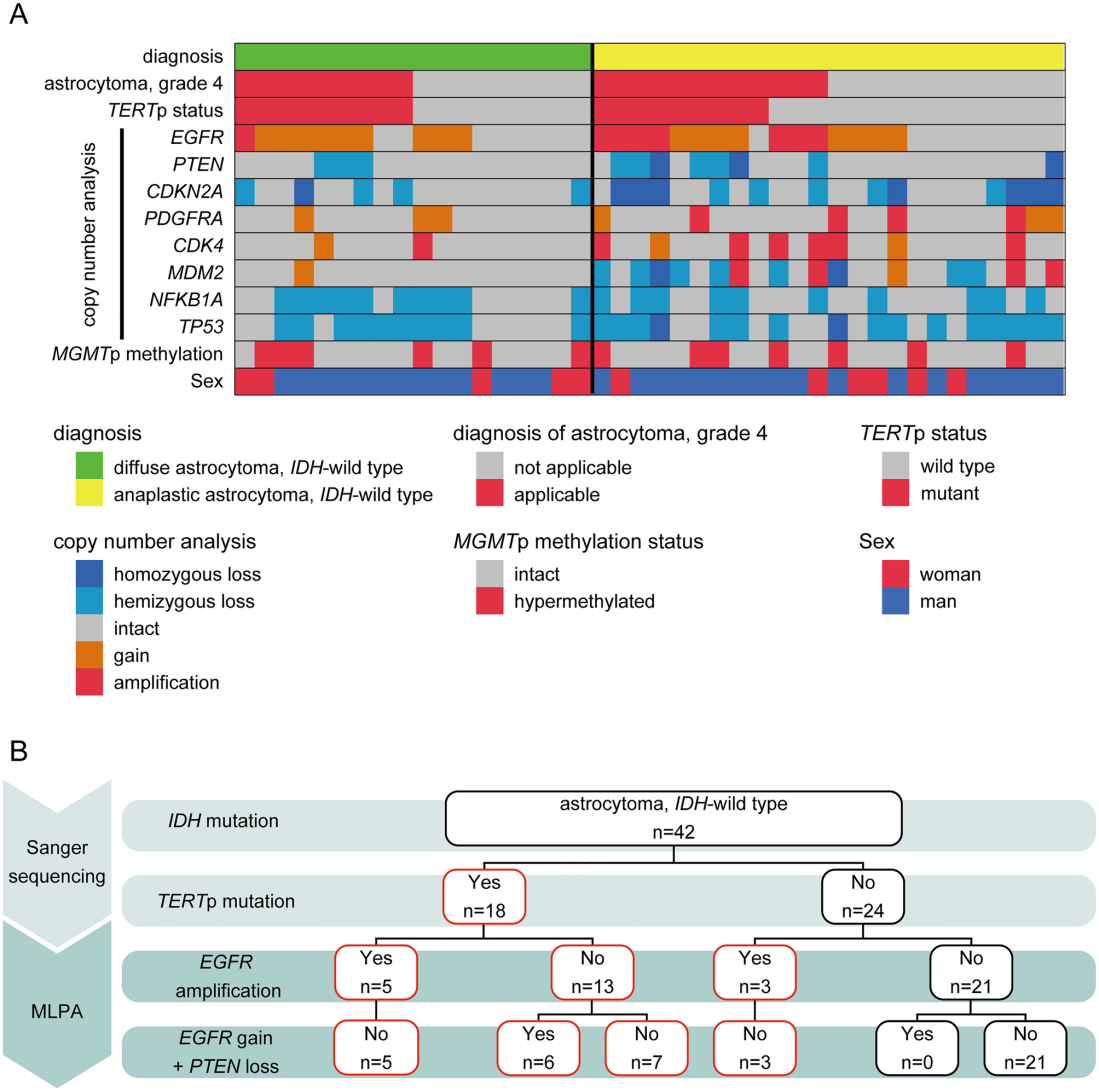
(**A**) The cell plot shows characteristics of all 42 *IDH*-wild-type astrocytomas. (**B**) The schema of three step classification of *IDH*-wild-type astrocytomas. *TERT*p mutation was examined by Sanger sequencing, and *EGFR* amplification and combination of *EGFR* gain and *PTEN* loss were examined by multiplex ligation-dependent probe amplification (MLPA). The case with red square was diagnosed as “astrocytoma. grade 4”.

The detailed status of mutations and alterations in the 42 *IDH*-wild-type astrocytomas is shown in Fig. 1-A. *TERT*p mutation was detected in 18 *IDH*-wild-type astrocytomas (42.9%), comprising 9 DAs (50%) and 9 AAs (37.5%). Again, no significant difference was seen between these two tumour types. By examination using MLPA KIT probemix P105, *EGFR* amplification was seen in 8 *IDH*-wild-type astrocytomas (19.0%), comprising 1 DA (5.6%) and 7 AAs (29.2%), tending to be slightly more frequent in AAs than in DAs (p = 0.109). The combination of *EGFR* gain and *PTEN* loss (+ *EGFR*/− *PTEN*) was detected in 6 *IDH*-wild-type astrocytomas (14.3%), comprising 3 DAs (16.7%) and 3 AAs (12.5%), showing no significant difference. All DAs with *EGFR* amplification or + *EGFR*/-*PTEN* showed *TERT*p mutation, so all DAs diagnosed as “astrocytoma, grade 4” were equivalent to DAs with *TERT*p mutation, while 3 *TERT*p-wild type AAs showed *EGFR* amplification. Interestingly, no astrocytomas with + *EGFR*/− *PTEN* lacked the *TERT*p mutation.

### Classification of lower-grade astrocytomas, IDH-wild type

The results of classification of IDH-wild-type lower-grade astrocytomas are shown in Fig. 1-B. As the third step of our classification system, the + *EGFR*/− *PTEN* classification was used as a surrogate marker for tumours with + 7/− 10. *TERT*p mutation was detected in 18 of all 42 *IDH*-wild-type astrocytomas, and these 18 tumours were classified as “astrocytoma, grade 4” from Sanger sequence alone. In the 24 tumours without *TERT*p mutation, MLPA showed *EGFR* amplification in 3 AAs, and no tumours with + *EGFR*/− *PTEN*. As a result, 21 of all 42 tumours were classified as “astrocytoma, grade 4”, comprising 9 DAs and 12 AAs, in the first and second steps of our system. In our cohort, all instances of “astrocytoma, grade 4” were able to be diagnosed by the combination of Sanger sequence and MLPA.

### Correlations with tumour profiles

Correlations between all pairs of the following factors were analysed: age at diagnosis, *TERT*p mutation, WHO grade, *O6-methylguanine-DNA methyltransferase* promoter (*MGMT*p) hyper methylation, and copy number alterations of *EGFR*, *PTEN*, *CDKN2A*, *PDGFRA*, *MDM2*, *CDK4*, *NFKBIA*, and *TP53*. *TERT*p mutation, *EGFR* amplification, *CDKN2A* homozygous loss, *CDK4* gain or amplification (gain/amplification), *CDK4* amplification, and *MGMT*p hypermethylation correlated with higher age at diagnosis (p = 0.0157, p = 0.0382, p = 0.0272, p = 0.0222, and p = 0.0045, respectively). Statistical correlations were detected between any two of *TERT*p mutation, *EGFR* gain/amplification and *PTEN* loss (*TERT*p and *EGFR* gain/amplification, odds ratio 5.91, p = 0.0236; *TERT*p mutation and *PTEN* loss, odds ratio 11, p = 0.004; *EGFR* gain/amplification and *PTEN* loss, odds ratio 9.38, p = 0.0304). Another correlation was seen between *PDGFRA* gain/amplification and *CDKN2A* homozygous loss (odds ratio, 7.78; p = 0.0195). WHO grade correlated significantly with *MDM2* loss and *MDM2* hemizygous loss (p = 0.0054 and p = 0.014, respectively). On the other hand, WHO grade showed no significance in the other profiles (Table 1).

### Survival analysis of lower-grade astrocytomas, IDH-wild type

**Figure 2:**
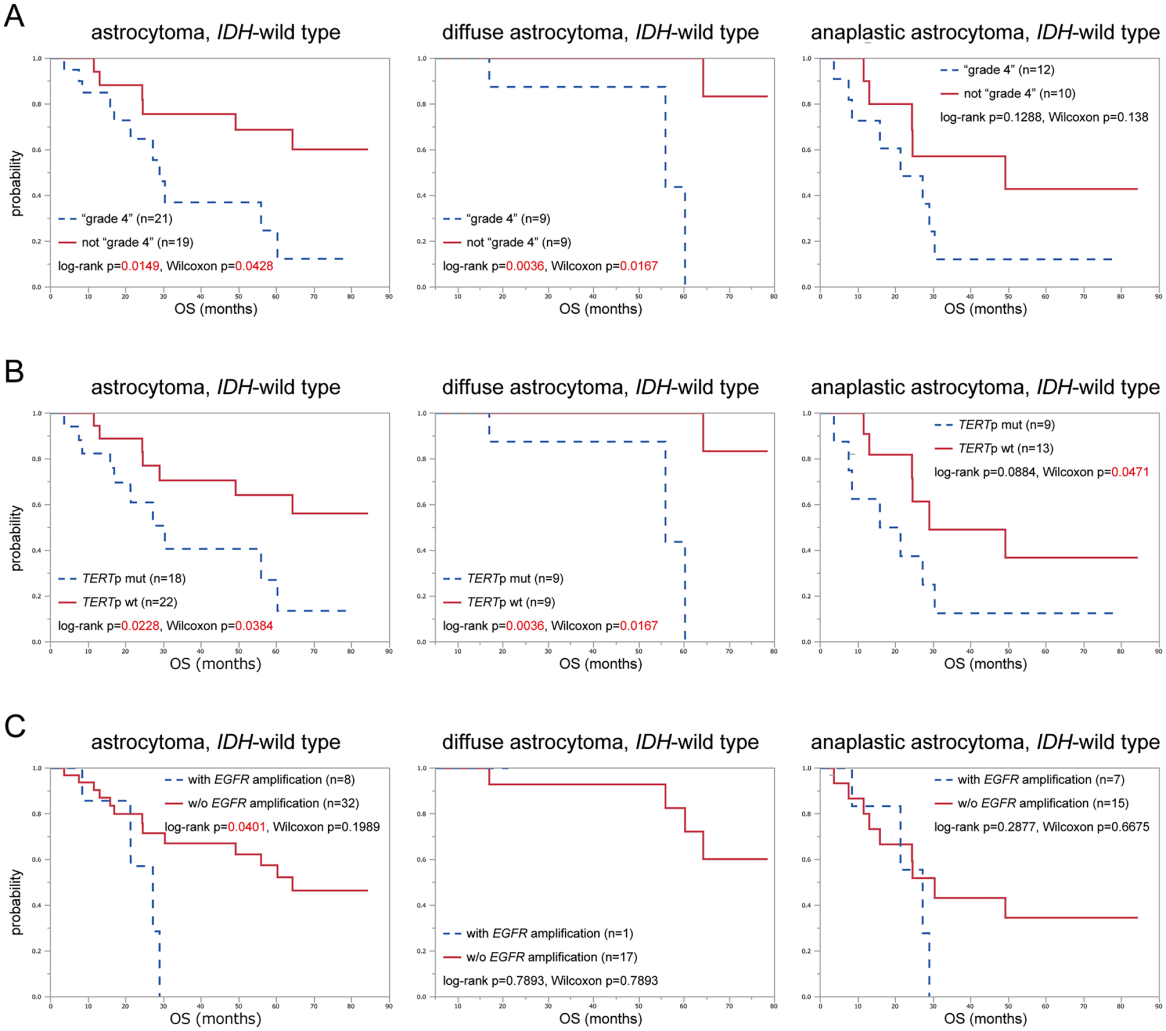
Survival analysis of 40 patients whose tumours were removed at their first surgery, were shown. The overall survival was analysed stratified by the following factors associated with the cIMPACT-NOW update 3 criteria; (**A**) the diagnosis of “astrocytoma, grade 4”, (**B**) *TERT*p mutation, or (**C**) *EGFR* amplification, in the groups of all *IDH*-wild type astrocytomas, *IDH*-wild type diffuse astrocytomas, and *IDH*-wild type anaplastic astrocytomas. The *p* values were calculated log-rank test and Wilcoxon test, and *p* < 0.05 was shown with red letters.

Clinical outcomes were calculated for the 40 cases in which the tumours were removed in first surgeries, including 18 DAs and 22 AAs. “Astrocytoma, grade 4” showed significantly shorter overall survival (OS) compared with other tumours in all astrocytomas (p = 0.0149) and DAs (p = 0.0036), but not in AAs (p = 0.1288) (Fig. 2-A). *TERT*p mutations were significantly associated with poor OS in all astrocytomas (p = 0.0228), and in DAs (p = 0.0036), but not in AAs (p = 0.0884) (Fig. 2-B). *EGFR* amplification was a significant factor for OS only in all astrocytomas (p = 0.0401), not in diffuse DAs (p = 0.7893) or AAs (p = 0.2877) (Fig. 2-C).

**Figure 3:**
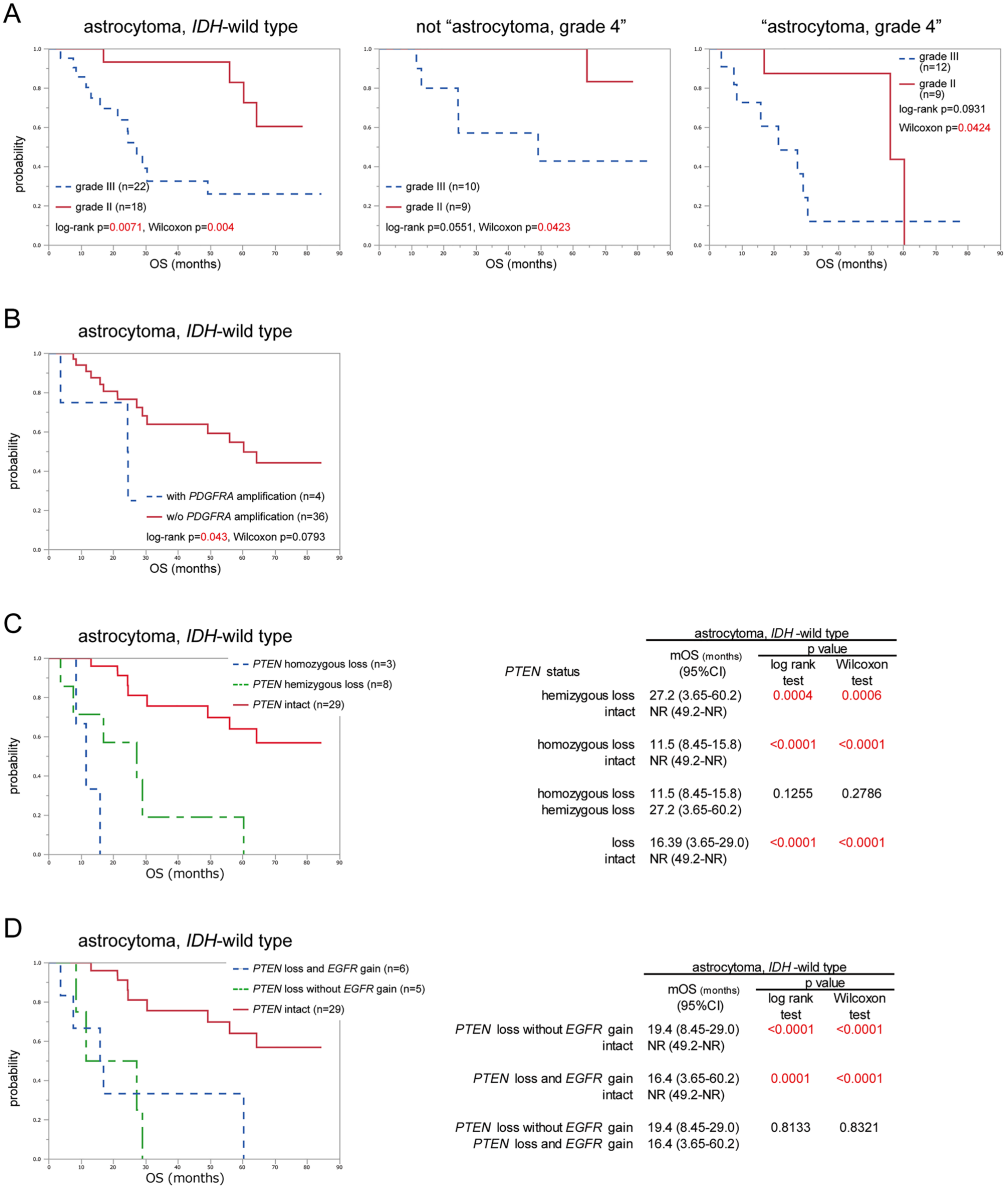
Survival analysis of 40 patients whose tumours were removed at their first surgery, were shown. (**A**) The overall survival of *IDH*-wild type diffuse astrocytomas (grade II) and anaplastic astrocytomas (grade III) were compared in the groups of all *IDH*-wild type astrocytomas, *“astrocytoma, grade 4”*, and not-“astrocytoma, grade 4”. The overall survival was also analysed stratified by the following factors associated with factors not included in cIMPACT-NOW update 3 criteria; (**B**) *PDGFR* amplification, (**C**) *PTEN* status (intact, hemizygous loss, or homozygous loss), and (**D**) the combination status of *PTEN* loss and *EGFR* gain. The p values were calculated log-rank test and Wilcoxon test, and p < 0.05 was shown with red letters.

The relationship between WHO grade and OS was also analysed, and AAs showed poorer prognosis than DAs (p = 0.007). For cases of “astrocytoma, grade 4” or “non-astrocytoma, grade 4”, AA showed poorer survival curves compared with DA, and significant differences were identified with the Wilcoxon test (p = 0.04 each), but not with the log-rank test (p = 0.09 and p = 0.055, respectively) (Fig. 3-A).

Log-rank testing showed significant correlations between OS and the following factors: age > 40 years, *EGFR* gain/amplification, *PDGFRA* amplification, *PTEN* homozygous loss and loss, *CDK4* gain/amplification, *MDM2* homozygous loss and alteration, and *TP53* homozygous loss (Table 2). These factors and *TERT*p mutation, *EGFR* amplification and the diagnosis of “astrocytoma, grade 4”, as described above, were analysed by Cox proportional hazard modelling. After the stepwise procedure, three factors remained significant: *PDGFRA* amplification with the largest risk ratio (risk ratio 13.9, p = 0.0022), *PTEN* loss (risk ratio 9.75, p = 0.0003) with the most significance, and *PTEN* homozygous loss (risk ratio 6.97, p = 0.0329) (Table 3). *PDGFRA* amplification was detected in 4 astrocytomas, and survival analysis showed a significant result (p = 0.043) (Fig. 3-B). *PTEN* loss was detected in 11 astrocytomas and 3 were homozygous losses and 8 were hemizygous losses, 6 of which were coincided with *EGFR* gains. A significant difference in OS was seen between astrocytomas with intact *PTEN* and those with *PTEN* loss (p < 0.0001), while there was no difference in OS between *PTEN* homozygous and *PTEN* hemizygous astrocytomas (p = 0.1255) (Fig. 3-C). No difference in survival of *PTEN*-loss astrocytomas was seen between presence or absence of *EGFR* gain (p = 0.8133), and *PTEN*-loss astrocytomas showed poor prognosis compared with *PTEN*-intact astrocytomas irrespective of whether *EGFR* gain was present (with *EGFR* gain, p = 0.0001; without *EGFR* gain, p < 0.0001) (Fig. 3-D).

**Table 2:** Results of Kaplan–Meier analysis and survival analysis for factors showing significance in survival analysis.

		n	Event	mOS (months) (95% CI)	p value	
					log-rank test	Wilcoxon test
cIMPACT-NOW update 3	“Grade 4"	21	11	29.0 (16.9–60.2)	0.0149	0.0428
	Not "grade 4"	19	6	NR (24.5–NR)		
*TERT*p	Mutant	18	10	30.4 (15.8–60.2)	0.0228	0.0384
	Wild type	22	7	NR (24.5–NR)		
*EGFR*	Amplification	8	4	27.2 (8.45–29.0)	0.0401	0.1989
	No amplification	32	13	64.2 (30.4–NR)		
	Gain/amplification	25	12	29.0 (21.3–60.2)	0.0227	0.0466
	Retained	15	4	NR (49.2–NR)		
*PDGFRA*	Amplification	4	3	24.4 (3.65–NR)	0.043	0.0793
	No amplification	36	14	60.2 (29.0–NR)		
*PTEN*	Homozygous loss	3	3	11.5 (8.45–15.9)	< 0.0001	< 0.0001
	No homozygous loss	37	14	60.2 (29.0–NR)		
	Lost	11	9	16.39 (3.65–29.0)	< 0.0001	< 0.0001
	Retained	29	8	NR (49.2–NR)		
*CDK4*	Gain/amplification	10	7	24.4(8.45–29.0)	0.0006	0.0125
	Retained	30	10	NR (49.2–NR)		
*MDM2*	Homozygous loss	2	2	16.4 (8.45–24.4)	0.008	0.022
	No homozygous loss	38	15	60.2 (29.0–NR)		
	Altered	15	9	24.5 (11.5–NR)	0.024	0.0279
	Retained	25	8	NR (49.2–NR)		
*TP53*	Homozygous loss	2	2	16.4 (8.45–24.4)	0.008	0.022
	No homozygous loss	38	15	60.2 (29.0–NR)		
Age	Age > 40 years	34	16	49.2 (24.4–64.2)	0.0345	0.0524
	Age ≤ 40 years	6	1	NR (30.4–NR)		
WHO grade	Grade III	22	13	27.2 (13.1–49.2)	0.0071	0.004
	Grade II	18	4	NR (55.9–NR)		

*CI* confidence interval, *NR* not reached, *mOS* median overall survival.

**Table 3:** Results of Cox proportional hazard model analysis of OS for each factor.

	Risk ratio (95% CI)	p value
WHO 2016, grade III	6.46 (0.942–44.4)	0.0575
“Astrocytoma, grade 4”	0.218 (0.00336–14.2)	0.4744
*TERT*p mutation	5.14 (0.129–204)	0.3838
*EGFR* amplification	0.709 (0.086–5.84)	0.7493
*EGFR* gain/amplification	3.04 (0.261–35.4)	0.3749
*PDGFRA* amplification	2.17 (0.0721–65.5)	0.6549
*PTEN* homozygous loss	1.65 (0.055–49.4)	0.7730
*PTEN* loss	5.93 (0.598–58.8)	0.1283
*CDK4* gain/amplification	1.16 (0.0755–17.7)	0.9166
*MDM2* homozygous loss	1.39 (0.186–10.4)	0.7467
*MDM2* alteration	1.47 (0.212–10.2)	0.6978
Age > 40 years	10.9 (0.372–319)	0.1657
**After stepwise procedure**		
*PTEN* loss	9.75 (2.82–33.7)	0.0003
*PTEN* homozygous loss	6.97 (1.17–41.5)	0.0329
*PDGFR* amplification	13.9 (2.58–75.2)	0.0022

The initial candidates (written above the bar) are all factors for which Kaplan–Meier analysis or log-rank testing showed significant results.

*CI* confidence interval.

### Validation of the status of chromosomes 7 and 10

As described above, MLPA KIT probemix P105 can detect CNA of EGFR at 7p.11.2 (*EGFR*(7p)) and PTEN at 10q.23.31 (*PTEN*(10q)). For validation of our classification system of the status of chromosomes 7 and 10 using MLPA, all samples were analysed using an additional two different MLPA KIT probemixes: chromosomes 7q by P370 and 10p by P303, respectively (Supplementary Fig. S1). Among six samples of showing + *EGFR* (7p)/− *PTEN* (10q) by P105 probemix, five samples showed both 7q gain by P370 and 10p loss by P303. We further examined all genome-level CNA analysis using a chromosomal microarray (CMA). A total of nine cases for which sufficient DNA was available were examined by CMA analysis to validate the results of CNA acquired by MLPA. Two cases showing both *EGFR*(7p)/7q gain and 10p/*PTEN*(10q) loss by MLPA system were confirmed to show + 7/− 10 by CMA (Supplementary Figs. S1, S2). One case that showed 7p(*EGFR*)/7q gain and 10q(*PTEN*) loss, but no 10p loss by MLPA system, was also confirmed to have + 7/− 10 by CMA (Supplementary Figs. S1, S2). These findings indicated that all 6 samples showing + *EGFR* (7p)/− *PTEN* (10q) by MLPA P105 probemix kit had features of + 7/− 10, suggesting that the status of + *EGFR* (7p)/− *PTEN* (10q) detected by MLPA system could be used as a surrogate marker for the + 7/− 10 phenotype.

## Discussion

In the present study, the Sanger sequencing method could detect most instances of “astrocytoma, grade 4”, with the addition of MLPA successfully identifying all cases. Correlations between *TERT*p mutation and 7p gain or 10q loss have already been reported^11^. In the present study, correlation analysis showed that *TERT*p mutation was associated with *EGFR* gain and *PTEN* loss, and + *EGFR*/− *PTEN* astrocytoma always accompanied *TERT*p mutation. This fact may result from a relationship between *TERT*p mutation, gain of chromosome 7, and loss of chromosome 10. The database of The Cancer Genome Atlas (TCGA)^6^ included 86 *IDH*-wild-type grade II or III gliomas, and *TERT*p status was examined in 56 cases. *TERT*p mutation was detected in 37 cases, and *EGFR* amplification was shown in 15 out of 37 *TERT*p mutant gliomas and in 4 of 19 *TERT*p-wild-type gliomas. The status of + 7/− 10 was evaluated in 55 of the 56 cases of *TERT*p mutant grade II or III glioma, with 27 cases showing + 7/− 10. Among these 27 cases, 12 cases showed both *TERT*p mutation and *EGFR* amplification, 14 cases showed *TERT*p mutation alone, and the remaining one case showed *EGFR* amplification alone. In the present study, *EGFR* amplification was defined as different from *EGFR* gain, so + *EGFR*/− *PTEN* tumours never showed *EGFR* amplification. As in the present study, for *IDH*-wild-type grade II or III gliomas in TCGA database, *TERT*p status revealed almost all cases of “astrocytoma, grade 4” and the addition of *EGFR* status successfully identified all other cases of “astrocytoma, grade 4”. Sanger sequencing and MLPA were thus thought to be reasonable methods for classifying *IDH*-wild-type lower-grade gliomas based on the recommendations from cIMPACT-NOW update 3^15^.

WHO grade offered a good marker of prognosis in the present study. *IDH*-wild-type AAs showed lower survival curve than *IDH*-wild-type DAs. Based on the WHO 2016 classification, glioma grade is partly affected by molecular factors including 1p/19q codeletion and histone mutations, but gliomas are mainly classified according to histological characteristics, which are almost the same as in the WHO 2007 classification. Although our cohort showed no differences in sex, age, or other prognostic factors detected in multivariate analysis, treatment selections did differ between DAs and AAs. Patients with AAs tended to be initially treated with strong chemoradiation therapies like the Stupp regimen^23^, and those with DAs tended to undergo observation alone more often; this might be one reason why the results of log-rank testing showing no statistical difference. The survival curve of DAs was definitely favourable compared with AAs in the early course, and the generalized Wilcoxon test showed a significant difference. Taking *IDH*-mutation status into consideration, WHO grade was reported as a significant factor for OS in lower-grade gliomas^11,24,25^, and one of the studies showed that WHO grade had higher prognostic value in *IDH*-wild-type astrocytomas compared with in *IDH*-mutant astrocytomas, with the authors proposing histological mitotic count as a significant predictor of prognosis^24^. This fact supports the importance of histological grading systems, especially for *IDH*-wild-type astrocytoma.

The survival analysis showed *TERT*p mutation as a prognostic factor for OS in the group of all *IDH*-wild-type astrocytomas and *IDH*-wild-type DAs, and the diagnosis of “astrocytoma, grade 4” with *EGFR* amplification was significant only in all *IDH*-wild-type astrocytomas. *TERT*p mutation and *EGFR* amplification have been reported as characteristics of *IDH*-wild-type GBM and as unfavourable prognostic factors in *IDH*-wild-type astrocytomas in many studies^10–12,26^, although a few studies have reported no significance^14,27^. In our study, *EGFR* amplification was slightly more frequent in AAs than in DAs, while *TERT*p showed no difference between subtypes. *TERT*p mutation, *EGFR* amplification, and diagnosis of “astrocytoma, grade 4” were significant factors in the group of all *IDH*-wild-type astrocytomas.

As mentioned above, *WHO* grade, *TERT*p mutation, *EGFR* amplification and diagnosis of “astrocytoma, grade 4” were good predictors of *IDH*-wild-type astrocytomas in Kaplan–Meier analysis, but Cox proportional hazard modelling detected no significance for OS in these factors. According to the Cox proportional hazard model of our cohort, copy number alteration of *PTEN* and *PDGFRA* amplification were significant predictors of OS.

CNA of *PTEN* was a strong predictor of prognosis, as demonstrated by both the Kaplan–Meier method and Cox proportional hazard modelling. No difference in OS was evident between *PTEN* hemizygous loss astrocytoma and *PTEN* homozygous loss astrocytoma, with both showing shorter OS than *PTEN*-intact astrocytoma. In addition, whether combined with *EGFR* gain or not, *PTEN* loss resulted in a significant difference in OS. *PTEN* loss is one of the typical genetic alterations in GBM, observed in about 30–40%^28,29^. Some studies of prognostic factors in GBM patients have been published, but the significance of *PTEN* loss has been controversial^27,30,31^. However, for *IDH*-wild-type lower-grade astrocytoma, some papers have stated that *PTEN* loss is associated with poor prognosis^27,32^, potentially because *PTEN* is a tumour suppressor gene^27,33^ and inactivation of *PTEN* signalling is thus important to malignant progression to glioblastoma^34^. The present study indicated *PTEN* loss as a strong predictor of poor prognosis in *IDH*-wild-type astrocytomas.

*PDGFRA* amplification showed a strong risk ratio in the present study, but only 4 AAs were included in the present study. *PDGFRA* amplification was also recognised as a characteristic of proneural GBM, which shows relatively good prognosis^29,35^. The frequency of *PDGFRA* amplification in lower-grade glioma has only been reported from studies of small numbers of low-grade gliomas^36,37^, and the evidence was insufficient to reach conclusions about the prognostic value. Strum et al. reported about subgrouping of GBMs based on the methylation profiles and compared them with other profiles of mutation and copy number status^35^. *PDGFRA* amplification was more common in a methylation cluster, “RTK I”, than in the other four clusters. “RTK I” cluster also showed *CDKN2A* loss frequently. In the present study, a correlation between *PDGFRA* gain/amplification and *CDKN2A* homozygous loss was seen, and might imply that astrocytoma with alteration of *PDGFR* is associated with “RTK I” GBM. In our cohort, no *PDGFRA* amplification was seen in DAs, but no difference in the frequency of this CNA was evident between DAs and AAs because of the small number with *PDGFRA* amplification. Further studies are required to clarify the prognostic value of *PDGFRA*.

After cIMPACT-NOW update 3, genetic analyses such as copy number analysis have been extensively studied for lower-grade glioma, and it has become clearer that several genetic markers are surely prognostic and need to be incorporated into clinical practice. To examine CNA at the whole-genome level, microarray systems or next-generation sequencing are generally used. However, these diagnostic systems require special equipment that carries a higher running cost. Those poor accessibility to them is usually unfavourable. In this context, our study showed important implications by showing that such prognostic stratification can be performed by direct sequencing and MLPA with simple methods at reasonable cost. The results of our validation study, all 6 tumours with + *EGFR*/− *PTEN* as determined by MLPA with P105 probemix showed + 7/− 10 in CMA or with additional MLPA methods. In these cases, one showed different results for 10p. In this case, CMA revealed 10p loss, but the copy number ratio as calculated by MLPA was 0.84, slightly higher than the threshold for chromosomal loss. Two cases with intact CNA as determined by CMA also revealed normal CNA by MLPA. Although further studies are essential regarding the results of MLPA and CMA for CNA in chromosome 7q or 10p, the results of CNA for *EGFR* and *PTEN* as detected by the MLPA system were suggested to offer good indicators for + 7/− 10.

Several limitations to the present study need to be considered. First, the study population was small. Second, the results of MLPA analysis were not able to be confirmed by other methodologies. Comparison of the results of MLPA and CMA were performed in nine cases due to the small amounts of DNA extracted from tumours. Third, the present study used only fresh or cryopreserved specimens, to obtain a sufficient quality and quantity of DNA and to avoid the influence of the paraffin embedding process to obtain precise results. Sanger sequencing and MLPA are generally available for the analysis of DNA extracted from FFPE samples. However, extraction of a sufficient quality of DNA from FFPE samples is well recognised as being not always easy and the quality of fragmented DNA in FFPE samples sometimes makes molecular analysis difficult. Such issues should be addressed in future research.

## Conclusion

The present study showed that the combination of Sanger sequencing and MLPA was sufficient to identify a subgroup of patients with poorer prognosis in *IDH*-wild-type lower-grade astrocytoma. These patients were safely considered to have “astrocytoma, grade 4” according to the cIMPACT-NOW update 3 criteria. Our data also identified *PTEN* loss and *PDGFRA* amplification as significant prognostic factors, and these genetic alterations are good candidates for an upcoming new classification. WHO grade is still useful to predict the clinical course of patients with *IDH*-wild-type gliomas.

## Methods

### Ethics approval and consent to participate

This study was carried out in accordance with the principles of the Declaration of Helsinki, and approval was obtained from the institutional review board at Kyoto University Hospital (approval number: G1124). Informed consent was obtained from the patients or the parents/ legally authorized representatives of subjects that are under 18 for inclusion in this study.

### Subjects

The purpose of the present study was to assess the feasibility of combining Sanger sequencing and MLPA in classifying *IDH*-wild-type lower-grade astrocytomas, as diagnosed by the WHO 2016 classification, into a new classification recommended by cIMPACT-NOW update 3, and to reveal prognostic factors for *IDH*-wild-type lower-grade astrocytoma.

The targets of the present study were *IDH*-wild-type astrocytomas surgically treated in Kyoto University Hospital. Inclusion criteria were as follows: (1) tumour samples after removal were stored as frozen or fresh specimens to maintain sufficient quality and quantity of DNA for extraction; (2) initial diagnosis was WHO grade II or grade III glioma; (3) Sanger sequence revealed no hot-point mutations in *IDH1/2*, *H3F3A*, or *HIST1H3B*; (4) MLPA showed no 1p/19q codeletion; and (5) informed consent was obtained.

### Sanger sequencing

Tumour DNA was extracted from tumour specimens using NucleoSpin Tissue (MACHEREY–NAGEL, Düren, Germany). Regions of interest for driver genes^3,16–18^ were amplified by PCR with gene-specific primers (Supplementary Table S1) and TaKaRa Ex Taq (TAKARA BIO, Shiga, Japan) (*IDH1/2*, *H3F3A*, and *HIST1H3B*) or AmpliTaq Gold 360 (Thermo Fisher Scientific, Waltham, MA) (*TERT*p) using an Applied Biosystems GeneAmp PCR System 9700 (Thermo Fisher Scientific). PCR products were processed by ExoSAP-IT (Thermo Fisher Scientific), then sequenced with sequencing primer (*IDH1*) or PCR forward primer as a sequencing primer (*IDH2*, *H3F3A*, *HIST1H3B*, *TERT*p) and a BigDye Terminator V1.1 Cycle Sequencing Kit (Thermo Fisher Scientific) using the ABI 3130xL Genetic Analyzer (Thermo Fisher Scientific).

### MGMT promoter methylation analysis

*MGMT*p methylation was assessed by quantitative methylation-specific PCR (qMSP), in accordance with previous reports^20,38^. Genomic DNA samples were processed using an EZ DNA Methylation Gold Kit (Zymo Research Corporation, Irvine, CA). Methylated and unmethylated molecules were quantified by qMSP using a QuantStudio 12 K Flex Real-Time PCR System (Thermo Fisher Scientific) with POWER SYBR Green Master Mix (Thermo Fisher Scientific) and specific primers (Supplementary Table S1)^39^ according to the standard curve method. The methylation status of samples was determined from the ratio of methylated molecules using the cut-off value at > 1%^20^.

### MLPA

Copy number analyses of 1p/19q, *EGFR*, *PTEN*, *CDKN2A* and *PDGFRA* were performed with MLPA according to the instructions from the manufacturer (SALSA MLPA KIT probemix P088-C2 for 1p/19q analysis and SALSA MLPA KIT probemix P105-D2 for the others; MRC-Holland, Amsterdam, the Netherlands)^19,20^. MLPA with probemix P105-D2 can also analyse the alteration of *MDM2*, *CDK4*, *NFKBIA*, and *TP53*. To determine the copy number status of chromosomes 7p and 10q, in the present study, gain of 7p and loss of 10q were substituted by *EGFR* gain and *PTEN* loss, which were determined by MLPA with probemix P105. The statuses of chromosomes 7q and 10p were analysed with SALSA MLPA KIT probemix P370-C1 and P303-A3 (MRC-Holland). The P370-C1 had 13 target probes in chromosome 7q and P303-A3 had 3 target probes in chromosome 10p.

Data on MLPA were collected using an ABI 3130xL Genetic Analyzer (Thermo Fisher Scientific), then analysed using Coffalyzer.Net Software (MRC-Holland). Thresholds of copy number detection were chosen as reported previously^19,40^, and set at 0.8–1.2. A ratio of 0.4 was set as the threshold between hemizygous and homozygous losses, and ratios > 2.0 were defined as amplifications.

### Integrated diagnosis

Tumour grading was performed histologically. Using all molecular pathological information, all cases received integrated diagnoses according to the 2016 WHO classification for central nervous system tumours^5^.

### Clinical outcomes

Clinical data retrospectively collected from electronic records included age at diagnosis, sex, treatment protocol as chemotherapy or radiotherapy, and dates of surgery, last follow-up, and death.

### Classification of astrocytomas, IDH-wild type

All *IDH*-wild-type astrocytomas were analysed by Sanger sequencing and MLPA to reveal the status of *TERT*p, *EGFR* gain or amplification, and *PTEN* loss. Classifications were performed in three steps. The first step was *TERT*p mutation, the second was *EGFR* amplification, and the third was + *EGFR*/− *PTEN*.

According to the recommendations of a previous study^15^, “astrocytoma, grade 4” was defined as *IDH*-wild-type astrocytoma with one or more of *TERT*p mutation, *EGFR* amplification, or + 7/− 10. However, showing the whole chromosomal alteration with MLPA (with SALSA MLPA KIT probemix P105-D2) is impossible. Because *EGFR* and *PTEN* are located on chromosomes 7 and 10, respectively, we hypothesised that tumours with + 7/− 10 must have + *EGFR*/− *PTEN*. In our classification system, + *EGFR*/− *PTEN* was therefore used as a criterion in the third step.

### Chromosomal microarray

Whole-genome level CNA analysis was performed with Cytoscan 750 K array (Thermo Fisher Scientific) according to the protocol provided by the manufacturer. Data were analysed using Chromosome Analysis Suite (ChAS) software (Thermo Fisher Scientific). The reference database included the Database of Genomic Variants (GRCh38/hg38).

### Statistical analysis

All statistical analyses were performed using JMP Pro version 15.1.0 software (SAS Institute, Cary, NC). Differences in categorical variables were evaluated using Fisher’s exact test or the chi-square test, and Student’s t-test was used for continuous variables. For survival analyses, OS was defined as the interval between the initial operative day and the date of death, or the last follow-up date on which the patient was known to be alive. Survival data were analysed using the Kaplan–Meier curve and the *p*-value for survival in the present paper was determined by log-rank testing if there was no special comment, while the generalized Wilcoxon test was used when appropriate. Multivariate analysis was performed by Cox proportional hazard modelling. Relationships between OS and the following factors were analysed: age; *MGMT*p hypermethylation; gain, amplification, and gain/amplification of *EGFR*, *PDGFRA*, and *MDM2*; hemizygous loss, homozygous loss and loss (including both of hemizygous and homozygous loss) of *CDKN2A*, *PTEN*, *CDK4*, *MDM2*, *NFKBIA*, and *TP53*. Differences were considered significant for values of *p* < 0.05.

## Supplementary Information

Supplementary Figure Legend.

Supplementary Table S1.

Supplementary Figure S1.

Supplementary Figure S2.

## Data Availability

The datasets used and/or analysed during the current study are available from the corresponding author on reasonable request.

## Abbreviations

AA: Anaplastic astrocytoma, grade 3
*CDK4*: Cyclin-dependent kinase 4
*CDKN2A*: Cyclin-dependent kinase inhibitor 2A
CI: Confidence interval
CNA: Copy number alteration
cIMPACT-NOW update 3: The Consortium to Inform Molecular and Practical Approaches to CNS Tumour Taxonomy
CMA: Chromosomal microarray
DA: Diffuse astrocytoma, grade 2
*EGFR*: Epidermal growth factor receptor
FFPE: Formalin-fixed paraffine-embedded
GBM: Glioblastoma
*H3F3A*: H3 histone, family 3A
*HIST1H3B*: Histone cluster 1 H3 family member b
*IDH*: Isocitrate dehydrogenase
*MDM2*: Murine double minute 2
MGMTp: O6-Methylguanine-DNA methyltransferase promoter
MLPA: Multiplex ligation-dependent probe amplification
*NFKBIA*: Nuclear factor kappa-B inhibitor alpha
OS: Overall survival
*PDGFRA*: Platelet-derived growth factor receptor A
*PTEN*: Phosphatase and tensin homolog deleted from chromosome 10
qMSP: Quantitative methylation-specific PCR
TCGA: The Cancer Genome Atlas
*TERT*: Telomerase reverse transcriptase
TERTp: Telomerase reverse transcriptase promoter
*TP53*: Tumour protein p53
WHO: World Health Organization
