# Supplementary material for: Prognostic stratification for IDH-wild-type lower-grade astrocytoma by Sanger sequencing and copy-number alteration analysis with MLPA
Source: Sci Rep. 2021 Jul 13;11:14408. doi: 10.1038/s41598-021-93937-8 (PMC8277860; doi:10.1038/s41598-021-93937-8)
Supplement: Supplementary file 1 — Supplementary Figure Legend. [file 41598_2021_93937_MOESM1_ESM.docx]

**Prognostic stratification for *IDH*-wild-type lower-grade astrocytoma by Sanger sequencing and copy-number alteration analysis with MLPA**

Yasuhide Makino^1,2^, Yoshiki Arakawa^*1^, Ema Yoshioka^2^, Tomoko Shofuda^2^, Takeshi Kawauchi^1,2^, Yukinori Terada^1^, Masahiro Tanji^1^, Daisuke Kanematsu^2^, Yohei Mineharu^1^, Susumu Miyamoto^1^, Yonehiro Kanemura^*2,3^

1) Department of Neurosurgery, Kyoto University Graduate School of Medicine, Kyoto, Japan

2) Department of Biomedical Research and Innovation, Institute for Clinical Research, National Hospital Organization Osaka National Hospital, Osaka, Japan

3) Department of Neurosurgery, National Hospital Organization Osaka National Hospital, Osaka, Japan

**Supplementary Figure S1**

The cell plot shows the status of copy number alterations (CNAs) of 42 cases.

The upper 4 lines show results made by multiplex ligation-dependent probe amplification (MLPA). The lower 4 lines are the results of 9 cases analysed by chromosomal microarray (CMA), these cases are named as #1-9 and their results are detailed in Supplementary Fig. S2 with the results of MLPA.

**Supplementary Figure S2**

The results of chromosomal microarray and MLPA of case #1-9 (described in Supplementary Fig. S1) are shown. The locations of EGFR (at 7p.11.2) and PTEN (at 10p.23.31) are marked by gray lines in the result of chromosomal microarray.
