# Supplementary material for: Prognostic stratification for IDH-wild-type lower-grade astrocytoma by Sanger sequencing and copy-number alteration analysis with MLPA
Source: Sci Rep. 2021 Jul 13;11:14408. doi: 10.1038/s41598-021-93937-8 (PMC8277860; doi:10.1038/s41598-021-93937-8)
Supplement: Supplementary file 2 — Supplementary Table S1. [file 41598_2021_93937_MOESM2_ESM.docx]

**Prognostic stratification for *IDH*-wild-type lower-grade astrocytoma by Sanger sequencing and copy-number alteration analysis with MLPA**

Yasuhide Makino^1,2^, Yoshiki Arakawa^*1^, Ema Yoshioka^2^, Tomoko Shofuda^2^, Takeshi Kawauchi^1,2^, Yukinori Terada^1^, Masahiro Tanji^1^, Daisuke Kanematsu^2^, Yohei Mineharu^1^, Susumu Miyamoto^1^, Yonehiro Kanemura^*2,3^

1) Department of Neurosurgery, Kyoto University Graduate School of Medicine, Kyoto, Japan

2) Department of Biomedical Research and Innovation, Institute for Clinical Research, National Hospital Organization Osaka National Hospital, Osaka, Japan

3) Department of Neurosurgery, National Hospital Organization Osaka National Hospital, Osaka, Japan

Supplementary Table S1. Primer list

|  | Forward primer | Reverse primer |
| --- | --- | --- |
| *IDH1* exon 4 [6] | 5'-AATGAGCTCTATATGCCATCACTG-3' | 5'-TTCATACCTTGCTTAATGGGTGT-3' |
| *IDH1* sequencing [6] | 5'-GCCATCACTGCAGTTGTAGGTTA-3' |  |
| *IDH2* exon 4 [40] | 5'-TTGTTGCTTGGGGTTCAAAT-3' | 5'-TGTGGCCTTGTACTGCAGAG-3' |
| *H3F3A* exon 1 | 5'-GATTTTGGGTAGACGTAATCTTCA-3' | 5'-TACATACAAGAGAGACTTTGTCCC-3' |
| *HIST1H3B* exon 1 | 5'-GGGCAGGAGCCTCTCTTAAT-3' | 5'-ACCAAGTAGGCCTCACAAGC-3' |
| *TERT*p [4] | 5'-TCCCTCGGGTTACCCCACAG-3' | 5'-AAAGGAAGGGGAGGGGCTG-3' |
| *MGMT*p qMSP-Unmet [17] | 5'-TTTGTGTTTTGATGTTTGTAGGTTTTTGT-3' | 5'-AACTCCACACTCTTCCAAAAACAAAACA-3' |
| *MGMT*p  qMSP-Met [17] | 5'-TTTCGACGTTCGTAGGTTTTCGC-3' | 5'-GCACTCTTCCGAAAACGAAACG-3' |
