# Supplementary figures and images for: Prognostic stratification for IDH-wild-type lower-grade astrocytoma by Sanger sequencing and copy-number alteration analysis with MLPA
Source: Sci Rep. 2021 Jul 13;11:14408. doi: 10.1038/s41598-021-93937-8 (PMC8277860; doi:10.1038/s41598-021-93937-8)

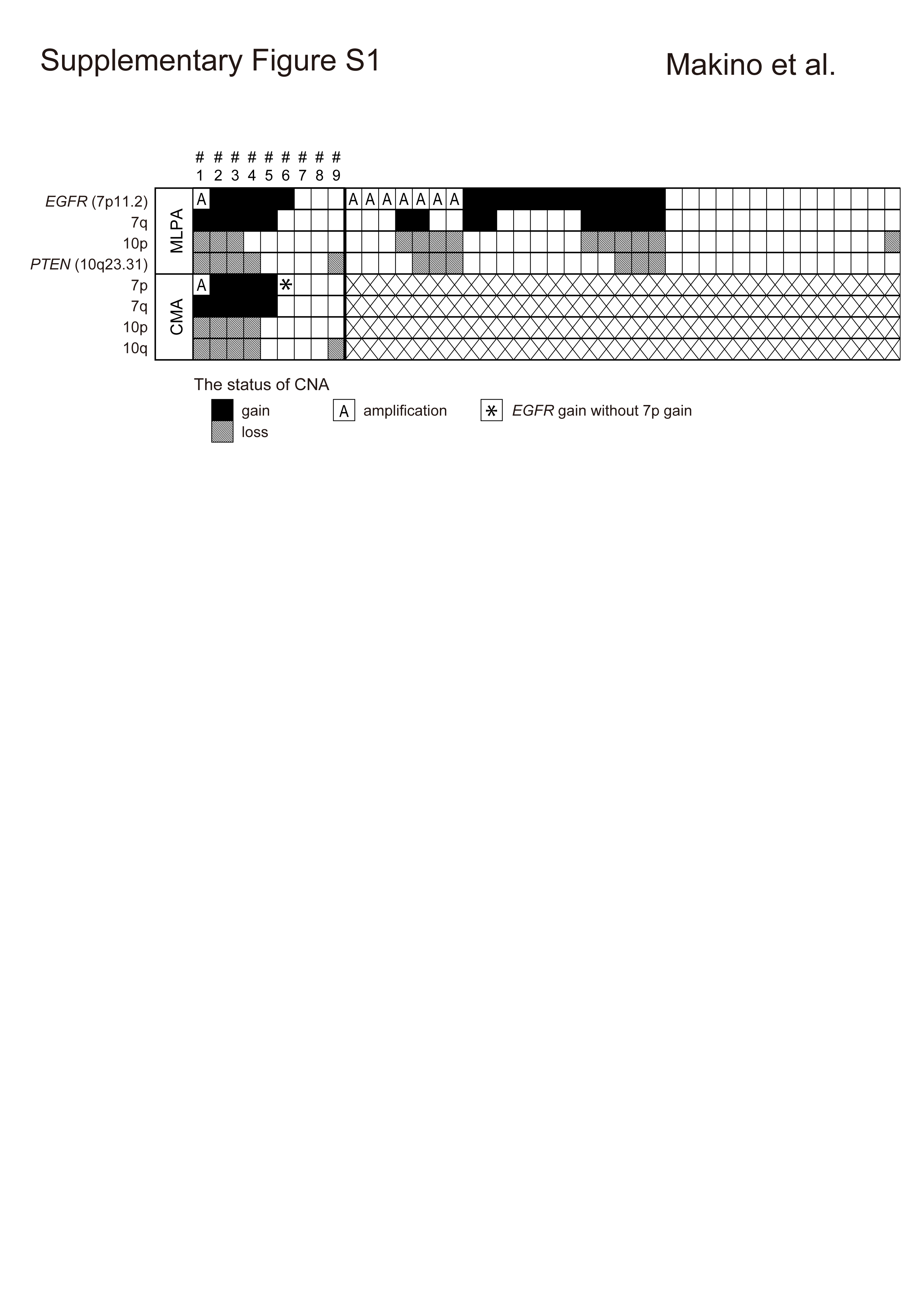

Supplement: Supplementary file 3 — Supplementary Figure S1. [file 41598_2021_93937_MOESM3_ESM.tif]

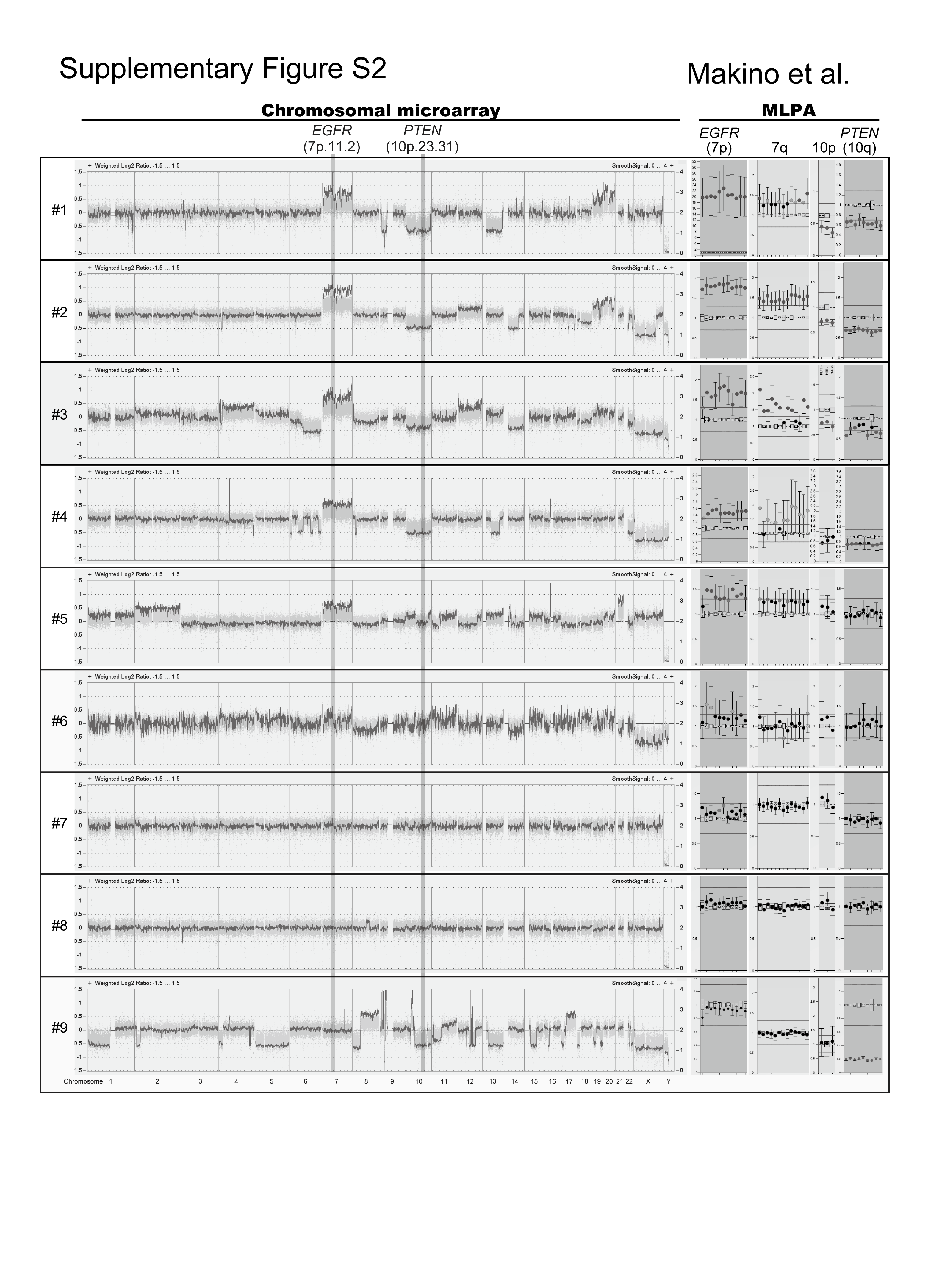

Supplement: Supplementary file 4 — Supplementary Figure S2. [file 41598_2021_93937_MOESM4_ESM.tif]
